# Cholera Infection Risks and Cholera Vaccine Safety in Pregnancy

**DOI:** 10.1155/2023/4563797

**Published:** 2023-05-22

**Authors:** Pamela El Hayek, Myriam Boueri, Leah Nasr, Christine Aoun, Edouard Sayad, Karl Jallad

**Affiliations:** ^1^Gilbert and Rose-Marie Chagoury School of Medicine, Lebanese American University, Lebanese American University School of Medicine, Lebanon; ^2^Department of Obstetrics and Gynecology, Lebanese American University School of Medicine, Lebanon; ^3^Department of Pediatrics, Division of General Pediatrics, Division of Pediatric Pulmonology, Lebanese American University Medical Center LAUMCRH, Beirut, Lebanon

## Abstract

**Introduction:**

Discuss the impact of cholera infection on pregnant women, fetus, and neonates and review the safety of cholera vaccines in pregnancy.

**Methods:**

This study was carried out as a narrative review during November 2022. A thorough literature review was conducted on the following databases: PubMed, Scopus, SciELO, CINAHL, Web of Science, and ScienceDirect. The following parameters were assessed from the included studies: type of cholera vaccine, cholera symptoms, cholera treatment, effect of cholera on pregnancy, effect of cholera treatment on pregnancy, effect of cholera vaccine on pregnancy, risk factors for fetuses and neonates, and prevention of cholera. The authors independently extracted data from the 24 included studies.

**Results:**

Cholera infection is a serious threat on pregnancy as it could lead to increased stillbirths and neonatal death. Fetal death was shown to occur mainly in the third trimester as most of the pregnant women infected with cholera had spontaneous abortions even after controlling for other confounding variables such as maternal age, dehydration level, and vomiting. Neonatal death was attributed mainly to congenital malformations and low Apgar scores with no improvements. Besides, cholera vaccines have shown to be safe in pregnancy and have proven to lower fetal and neonatal malformations among vaccinated compared to nonvaccinated pregnant women.

**Conclusion:**

This narrative summarizes the different complications due to cholera infection in pregnancy. It also reviews the safety of cholera vaccine administration in pregnant women.

## 1. Introduction

Cholera is a serious infectious disease caused by toxigenic serogroups O1 and O139 of *Vibrio cholerae*, a curved Gram-negative bacilli. Fecal contamination of water sources and food is the main route of transmission of this disease [1]. Via activating adenylate cyclase, cholera toxin (CT) is responsible for the massive loss of chloride ions and other electrolytes through a typical diarrhea whose consistency is described as “rice watery stools” [1]. It is estimated that cholera is endemic in almost 50 countries with the majority of cases spread in Asia and Africa [2]. Even though the majority of the infected population have a mild to moderate disease, cholera can lead to severe dehydration and death [3]. A mortality rate exceeding 50% has been associated with untreated cholera infection [4].

Cholera is a major public health concern since it does not only cause outbreaks or epidemics in a specific vulnerable country but can also spread to the surrounding area, spanning the entire region [5]. Vulnerable individuals, such as pregnant women, infants, elderly, immunocompromised patients, and people with low socioeconomic status, are at a higher risk of developing a life-threatening cholera infection [6]. The severity of cholera infection during pregnancy has been recognized, as early as the nineteenth century [7]. Similarly, current literature acknowledges the adverse outcomes of cholera infection on pregnancy [8]. Having said that, pregnant women are of major concern due to the high fatality rate associated with cholera infection during pregnancy and multiple serious fetal outcomes such as abortion and prematurity [9]. Not only is cholera associated with death but also with serious effects on fetal growth. Ogasawara and Inoue found that cholera exposure and height stunting are proportional [10]. Therefore, it has become of interest not only to treat infected pregnant women but also to prevent them from getting infected.

Preventing cholera infection has been widely tackled in the literature, with clean water and sanitation being the mainstay of prevention. A well-known cholera prevention strategy termed water, sanitation, and hygiene (WASH) is providing safety. However, the global achievement of WASH still requires many years especially that endemic areas do not prioritize such program leading to an inadequate cholera prevention [11]. Additionally, implementing cholera prevention strategies is hard in countries where healthcare is underprioritized and conflicts are common [5]. With that being said, vaccines are the other tool needed for cholera prevention. Three oral cholera vaccines (OCVs) are widely used: Shanchol and Euvichol, which are bivalent killed vaccines derived from *Vibrio cholerae* O1 and O139, and Dukoral, a monovalent killed vaccine derived from *Vibrio cholerae* O1 and its recombinant toxin B subunit [12].

However, the lack of data about the safety of administering vaccines in pregnant women resulted in excluding pregnant women from mass vaccination campaigns [13]. Furthermore, pharmaceutical companies express their uncertainty about the safety of administering the vaccine to pregnant women [14]. That being said, a major ethical dilemma arose. This dilemma lies between the risks and benefits of administering a vaccine that might be harmful to pregnant women and their fetuses and withholding a lifesaving prevention tool [14]. Given the ongoing public health concern about cholera infection during pregnancy and the safety of administering OCVs to pregnant women, a review is needed to discuss the current literature. Our review summarizes the main literature findings regarding the severity of cholera during pregnancy, its outcomes, its effect on fetuses and neonates, and whether OCVs are safe to administer during pregnancy.

## 2. Methods

### 2.1. Search Strategy and Screening Process

**Figure 1:**
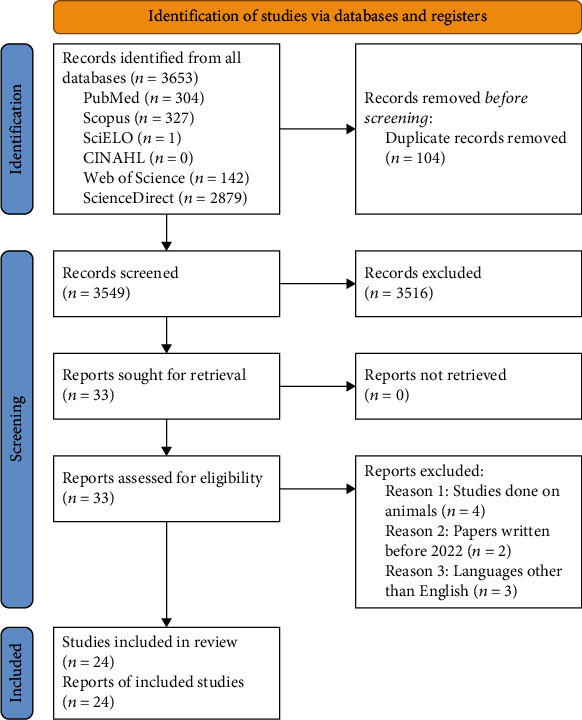
The PRISMA flow diagram of literature screening for cholera during pregnancy.

This study was carried out as a narrative review during November 2022. Institutional Review Board was exempt given that it poses no risk on patients. The authors performed a search through PubMed, Scopus, SciELO, CINAHL, Web of Science, and ScienceDirect using the following keywords: “Choleras”, “Vibrio cholerae Infection”, “Infection Vibrio cholerae”, “Infections Vibrio cholerae”, “Vibrio cholerae Infections”, “cholera”, “Cholera outbreak”, “cholera endemic”, “cholera epidemic”, “cholera pendemic”, “Pregnancy”, “Fetal complications”, “fetal death”, “neonatal mortality”, “maternal mortality”, “Gestation”, “pregnant women”, “stillbirth”, “abortion”, “retained fetus”, “fetal tissue”, “aborted fetus”, and “fetal complications”. The search was restricted to English articles published from 2002 till 2022. Case control, cross-sectional studies, cohort studies, clinical trials, and review articles about cholera during pregnancy were included (Figure 1).

Two authors performed the literature searches independently based on inclusion and exclusion criteria, deleting irrelevant literature, and abandoning duplications through Zotero. The results were then aggregated on Rayyan where three authors performed the screening process of the titles and abstracts. The following parameters were assessed from the included studies: type of cholera vaccine, cholera symptoms, cholera treatment, effect of cholera on pregnancy, effect of cholera treatment on pregnancy, effect of cholera vaccine on pregnancy, risk factors for fetuses and neonates, and prevention of cholera. Four authors independently extracted data from the included studies.

## 3. Results and Discussion

### 3.1. Search Results

By applying the PRISMA guideline, 3,653 articles were found throughout the different databases and stored on Zotero. A total of 104 duplicates were automatically detected and removed, and the remaining 3,549 articles were stored on Rayyan for further screening. A sum of 33 papers fulfilled the eligibility criteria and were then assessed for a full-text review inclusion. After a careful investigation, the authors excluded 4 studies done on animals, 2 papers written before 2022, and 3 papers in languages other than English. Hence, 24 articles were covered and discussed in this review. The screening process is better visualized through Figure 1.

### 3.2. Cholera during Pregnancy

In cholera endemic areas, pregnant women, as the rest of the general population, are at risk for severe episodes of diarrhea which can result in dehydration and impact the pregnancy course [5, 15–17]. In comparison with studies from 1969 to 1990, a meta-analysis of studies published from 1991 to 2013 showed a significantly lower absolute risk of maternal death related to cholera attributed to the introduction of oral rehydrating solutions (ORS) in the late 1970s for fluid and electrolyte replenishment [8], whereas, in the published Haiti cohort, no maternal death due to cholera or obstetric complications was reported despite the infection having an overall case-fatality rate of 2.25% in Haiti in 2010 [18] (Table 1).

Cholera infection has the greatest effect on fetal survival. In fact, in the 2013 Haiti study, an increase in 2- to 6-fold in fetal deaths in pregnant women infected with cholera was seen between 1969-1990 and 1991-2013 (adjusted risk ratio (RR) 5.7) with 21 (8%) pregnancies of 263 hospitalized pregnant women ending with intrauterine fetal deaths [8, 18]. Among the 900 pregnancies in the 2011 Haiti study, 141 (16%) fetal deaths occurred [17]. In the 3 regions of Haiti, Senegal, and Peru, the fetal death rate was 3.8 (95% CI: 2.1–7.1) times higher compared to national stillbirth estimates [19]. Besides, women infected with cholera are at a 6-fold higher risk of miscarriage and a 3-fold higher risk of stillborn child compared to women not infected with cholera [15]. Fetal deaths mostly occurred in the third trimester [5], and in the 1960's Bangladesh study, half of the pregnant women infected with cholera had spontaneous abortions in the third trimester [13] even after controlling for other confounding variables such as maternal age, dehydration level, and vomiting; third trimester fetal deaths were the highest which could actually be explained by the increase in the need of placental blood flow in later trimesters [17]. However, in the 2013 Haiti study, 8/21 fetal deaths occurred in the third trimester and 10/21 occurred in the second trimester [18] whereas in the 2015 meta-analysis, no difference in the risk of fetal death by trimester was indicated [8] (Table 1).

Fetal death is the result of physiologic abnormalities in the women infected with cholera [5]. As such, the strongest risk factors for fetal death in pregnant women infected with cholera were severe maternal dehydration and vomiting. In fact, out of 21 fetal deaths in the 2013 Haiti study, dehydration levels were recorded for 7 patients at the time of fetal death, among which 1 was severely dehydrated, 5 were moderately dehydrated, and 1 was not dehydrated [18]. Even after adjusting for confounding variables, dehydration levels were the strongest risk factors for fetal death in cholera-infected women (adjusted risk ratio (RR) for severe vs. mild dehydration is 9.4 (95% CI 2.5–35.3, *p* = 0.005)) [18]. Maternal dehydration due to cholera infection can lead to severe hypovolemia. This will compromise the perfusion of the placenta, hence, the fetus, and eventually lead to fetal death in utero especially when rehydration therapy is delayed due to fetal hypoxia and acidosis [15, 17, 18]. Unfortunately, even with aggressive rehydration therapy, fetal deaths were not prevented. Spontaneous abortions were twice as likely seen due to severe vomiting [17] which was another significant risk factor for fetal death independently of the severity of dehydration (RR 5.1, 95% 1.1–23.8, *p* = 0.041). In fact, electrolyte changes in the amniotic fluid [5] through the loss of bicarbonate contributes to maternal acidosis and hence is implicated in fetal death which was seen mainly in pregnant women with diabetes type 1 with ketoacidosis with a fetal mortality of 35% [18]. The cholera toxin is not the cause of fetal death per se; however, it leads to the secretory diarrhea of cholera-infected pregnant women which led to their severe dehydration and compromised fetal health, thereby increasing fetal death rates [18] (Table 1).

Neonatal outcomes did not show a higher mortality risk for neonates of pregnant women infected with cholera. The 2015 meta-analysis presented a significantly higher absolute risk of neonatal death in 1969-1990 compared to 1991-2013 [8]. Actually, in the 2013 Haiti study, among the 16 (6%) cholera-infected women who delivered a live baby of 35-40 weeks of gestation, 2 neonatal deaths occurred; one was attributed to congenital malformations and necrotic enteritis and the second death was correlated with an Apgar score of 2 with no further improvements, both of which were not associated with maternal cholera infection [18] (Table 1).

### 3.3. Prevention of Cholera + Vaccines in General

After discussing the effect of Cholera infection on pregnant women and specifically on the fetus and the neonate, it is crucial to discuss the means to prevent cholera infection. According to the World Health Organization (WHO), the mainstay of prevention of cholera endemic and outbreaks stems with water, sanitation, and hygiene also known as “WASH” practices which imply constructing access to clean potable water and adequate sanitation and hygiene measures [1]. In addition to that is the creation of prevailing sanitation measures and laws for food industries and vendors, such as promoting proper handwashing with soap and appropriate food handling. In fact, WASH implementation has proved through history its importance in elimination of cholera infections from Central and South America to Thailand and South Africa and to Tanzania and Zambia [5]. According to the Cholera Hospital-Based Intervention for 7 Days (CHOBI-7), a randomized controlled trial done in Bangladesh, households who applied the proper handwashing and water treatment measures had 41% fewer secondary cholera infections than households who did not receive these hygiene interventions [23] (Table 2).

However, the current WASH guidelines vary and are even in discordance [24]. Out of 95 recommendations, only 20 were consistently found in different guidelines with advancement in water and food hygiene being the most consistent recommendation [24]. These guidelines are considered within household transmission and community transmission with the majority of them discussing the community transmission strategies [24]. Concerning other limitations of the WASH guidelines, the extent of service needed to stop cholera in community transmission has not been specified. Additionally, the presence of numerous recommendations does not indicate their utility. Hence, focused and evidence-based guidelines are needed for the adequate prevention and control of cholera outbreaks and epidemics. Five strategies were proposed by D'Mello-Guyett et al. in order to improve guidelines validity and strength [24] (Table 3).

A complementary tool to prevent cholera infections and outbreaks is the administration of cholera vaccines especially in endemic countries and areas at high risk of outbreaks [25]. To date, 4 different types of cholera vaccines have been designed, licensed, and qualified by WHO [25]. Starting with the first internationally licensed oral cholera vaccine, the originally produced killed whole-cell and recombinant cholera toxin B vaccine [26]. The second cholera vaccine is the killed whole-cell only vaccines as it was manufactured without the cholera toxin B to reduce cost and complexity of the vaccine for administration [26]. The third type of vaccine produced is the live attenuated oral cholera vaccine that mimics the natural cholera infection by inducing mucosal gut immune response [25]. The fourth and final type of cholera vaccine is the parenteral cholera vaccines which are parenterally killed whole-cell cholera vaccines. These vaccines were only administered for a short period of time due to their side effects in the recipients, limited protection for a short duration, and their inability to limit transmission of cholera [25] (Table 4).

### 3.4. Cholera Vaccination during Pregnancy

Since 2009, several oral cholera vaccines (OCVs) have been provided to citizens above 1 year of age in low-income cities in Bangladesh. At first, vaccination campaigns omitted pregnant women due to the uncertainty of the safeness of those vaccines. However, after several retrospective and prospective studies being conducted, Khan et al. confirmed that there is no increased risk on the fetuses of OCV-exposed pregnant women [12]. On the contrary, pregnant women are encouraged to take this vaccine especially in regions highly susceptible to cholera [12] (Table 5).

For instance, in 2009, 196 pregnant women residing in Zanzibar were given the Dukoral vaccine. After a period of 9 months, birth surveillance was assessed to see whether any of those women had any birth complication including miscarriages or live birth [14]. Compared to 955 unvaccinated pregnant women, there was no significant fetal or birth complication. In fact, both groups had the approximately the same probability of spontaneous abortions, fetus abnormalities, or deaths [14] (Table 5).

Two years later, another study conducted by Khan et al. confirmed the results in the city of Dhaka. Despite limiting the vaccination campaign to 269,000 citizens excluding pregnant women, some were able to disregard the criteria and took the second dose of OCV while being pregnant. A study was done on few pregnant women reflecting the previous results and concluded that the OCV is safe for pregnant women and does not offer any risk to the fetus [12] (Table 5).

Khan et al. did not limit their study to Dhaka; in fact, 1,543 pregnant women in Guinea were also assessed in 2013 for taking the Shanchol vaccine during a campaign. This retrospective study demonstrated a negative relationship between the vaccine exposure and fetal complications [12]. Regardless of the vaccination, both groups of women were at equal risk of pregnancy loss, fetus death, and fetal abnormality. On a side note, it was shown that pregnant women were more concerned about the vaccine and were more likely to follow the instructions provided to them [12] (Table 5).

Nonetheless, another retrospective cohort study was done in Guinea to assess the effect of the bivalent killed whole-cell oral cholera vaccine (BivWC) on 2,494 pregnant women. 3.7% (95% CI 2.7–4.8) of miscarriages were observed among vaccinated women compared to 2.6% among nonvaccinated ones [20]. Moreover, vaccinated women were at lower risk of malformation frequency 0.6% (0.1-1.0) compared to nonvaccinated women 1.2% (0.0-2.5). Despite those results, Grout et al. concluded that even if there was a correlation between the exposure of the vaccine and the risk of miscarriage or fetal deformity, the risk would be negligible [20]. Like the previous listed study, women were more concerned about the information delivered to them and less occupied by other events. Hence, raising awareness on this issue was easily done. Apart from that, Ciglenecki et al. and Khan et al. had previously confirmed that women who were previously exposed to cholera had a higher chance of pregnancy loss and fetal abnormality compared to healthy women [12, 18]. Likewise, women in this retrospective cohort study were at a six times higher risk of developing those problems. Hence, vaccinating pregnant women has several advantages [27] (Table 5).

Two years following Grout et al.'s study, Ali et al. conducted a cohort study in Malawi during a cholera outbreak to observe whether the Shanchol vaccine was safe to be used for pregnant women [13]. Compared to 835 nonvaccinated women, 835 vaccinated women were at an equal risk of stillbirth, spontaneous abortion, birth defects, or fetal mortality [13] (Table 5).

Based on all the previously mentioned studies, the World Health Organization (WHO) released a position paper in 2017 stating the effectiveness and safeness of vaccinating all pregnant women against the cholera vaccine [1]. According to WHO [28] guidelines and recommendations, a summary of the risks and benefits of administering the different types of cholera vaccines in pregnant women is listed in Table 6 [28].

## 4. Limitations

The above results of our study are subject to the inherent limitations of a narrative review. Also, it is important to consider that articles from other databases or in languages other than English were not included. Finally, all articles containing animal models were disregarded which might have affected the results.

## 5. Conclusion

Cholera epidemics continue to threaten the lives of patients of all ages and particularly vulnerable population notably pregnant women, neonates, children, and immunocompromised patients. As the literature suggests, severe dehydration caused by cholera infection could affect the pregnancy course and lead to devastating outcomes for the mother and baby. Stillbirth and spontaneous miscarriages were reported following severe maternal dehydration and diarrhea due to cholera infection. Vaccines are shown to be safe and effective in pregnancy. Lastly, WASH implementation has demonstrated to be successful in eradicating cholera infection in endemic areas. Means to apply the strategies of WASH are yet to be administered globally due to demographic and financial obstacles. In addition, WASH guidelines vary and are found to be in in discordance, as some strategies have not been specified. Hence, focused and evidence-based guidelines are needed for the adequate prevention and control of cholera outbreaks and epidemics. Multiple strategies were proposed to improve guidelines validity and strength such as tackling human-to-human and environment-to-human transmissions of cholera instead of household and community transmissions and develop evidence-based guidelines by standardized models.

## Figures and Tables

**Table 1:** Outcomes and risk factors of cholera during pregnancy based on included studies.

Author	Country	Outcomes of cholera in pregnancy	Risk factors of cholera in fetus
Ciglenecki et al. [18], Schillberg et al. [17]	Haiti	86% of pregnant women were discharged with a preserved pregnancy, and 6% had live full-term singleton births, of whom 2 died within the first 5 days postpartum. The remaining pregnancies (8%) resulted in intrauterine fetal death [18]	The strongest risk factor for fetal death was severe maternal dehydration, followed by severe vomiting Risk factors for complications of cholera in pregnant women were third trimester, younger maternal age, severe dehydration, and vomiting [17]

Nguyen-Toan Tran et al. [8]	Switzerland	The pooled maternal death rate for 1991-2013 studies was 0.2% (95% CI: 0.0-0.7) and 5.0% (95% CI: 0.0-16.0) for 1969-1990	The pooled fetal death rate for 4 studies during 1991-2013 was 7.9%, significantly lower than that of 3 studies from 1969 to 1990

Grout et al. [20], Moro et al. [16], Ali et al. [13], Davis et al. [5], and Wierzba et al. [19]	USA	Pregnant women are at risk of severe diarrheal disease that can result in dehydration and pregnancy loss [16] They are at risk of premature childbirth, or maternal death if the patient is not treated properly [13], fetal acidosis and hypoxia [5], and fetal distress and death [19]	Fetal malformations

Zhang et al. [21]	China	NA	There was no association of pregnant women infected with cholera with an increased risk of preterm delivery, low birthweight, accidental abortion, or malformation

Khan et al. [15], Khan et al. [12]	Bangladesh	Pregnancy loss with magnitude varying from 2 to 36% Adverse outcomes of pregnancy with infection of cholera	No association

Hashim et al. [14]	Korea	Abortions, premature childbirth, and maternal death	No association

Friedrich et al. [22]	Malawi	Miscarriage and stillbirths	No association

**Table 2:** Recommendations of the water, sanitation, and hygiene (WASH) strategy.

1. Constructing access to clean potable water
2. Adequate sanitation and hygiene measures
3. Sanitation measures and laws for food industries and vendors, such as promoting proper handwashing with soap and appropriate food handling

**Table 3:** Proposed strategies for WASH guidelines improvement.

Tackle human-to-human and environment-to-human transmissions of cholera.
Improve guidelines: fewer but more detailed.
Detail each recommendation: response time and degree of services needed.
Encourage publishing and literature access.
Develop evidence-based guidelines via standardized models.

**Table 4:** Types, components, and characteristics of cholera vaccines.

Cholera vaccines	Components of cholera vaccines	Characteristics of cholera vaccines
Type I (Dukoral ®)	Killed whole-cell and recombinant cholera toxin B vaccine	First internationally licensed oral cholera vaccine

Type II (ORC-Vax™/mORC-Vax™, Shanchol™, Euvichol®, OraVacs)	Killed whole-cell only vaccines	Manufactured without the cholera toxin B to reduce cost and complexity of vaccine administration

Type III (CVD 103, Peru-15, Cuban 638, VA 1.4)	Live attenuated oral cholera vaccine	Mimics the natural cholera infection by inducing mucosal gut immune response

Type IV	Parenteral, killed whole-cell cholera vaccines	Administered for a short period of time due to their side effects in the recipients, limited protection for a short duration, and their inability to limit transmission of cholera

**Table 5:** Cholera vaccination during pregnancy results.

Author	Country	Effect of cholera vaccine on pregnancy
Hashim et al. [14]	Korea	Both groups had approximately the same probability of spontaneous abortions, fetus abnormalities, or deaths

Ciglenecki et al. [18]	Haiti	Women who were previously exposed to cholera had a higher chance of pregnancy loss and fetal abnormality compared to healthy women

Grout et al. [20]	USA	(i) 3.7% (95% CI 2.7–4.8) of miscarriages were observed among vaccinated women compared to 2.6% among nonvaccinated ones (ii) Vaccinated women were at lower risk of malformation frequency 0.6% (0.1-1.0) compared to nonvaccinated women 1.2% (0.0-2.5) (iii) Even if there was a correlation between the exposure of the vaccine and the risk of miscarriage or fetal deformity, it is negligible (iv) The OCV is safe for pregnant women and does not offer any risk to the fetus (v) A negative relationship was seen between the Shanchol vaccine exposure and fetal complications

Ali et al. [13]	USA	Compared to 835 nonvaccinated women, 835 vaccinated women were at an equal risk of stillbirth, spontaneous abortion, birth defects, or fetal mortality

Khan et al. [12]	Bangladesh	There is no increased risk on the fetuses of OCV-exposed pregnant women

WHO [1]	Worldwide	Vaccinating all pregnant women against the cholera vaccine is effective and safe

**Table 6:** Risks and benefits of cholera vaccines in pregnancy.

Type of vaccine	Risk	Benefit
OCV	No significant increase in adverse maternal or pregnancy outcomes	A decrease in cholera incidence
Dukoral	No adverse effects in pregnant women	NA
Shanchol	No adverse effects in pregnant women	74% protection to adults (age 15 and older) over a five-year period
Euvichol	No adverse effects in pregnant women	A strong vibriocidal response

## Data Availability

The 24 articles that were retrieved and data that was extracted are found in this google document: https://docs.google.com/spreadsheets/d/1_hozW-G7vICcpXXCMMIz4faBzpppZRGP6a-aWd39rGc/edit#gid=0.
